# The Correlation Between Circulating Ghrelin and Insulin Resistance in Obesity: A Meta-Analysis

**DOI:** 10.3389/fphys.2018.01308

**Published:** 2018-09-21

**Authors:** Cai-Shun Zhang, Liu-Xin Wang, Rui Wang, Yuan Liu, Li-Min Song, Jun-Hua Yuan, Bin Wang, Jing Dong

**Affiliations:** ^1^Department of Special Medicine, Medical College, Qingdao University, Qingdao, China; ^2^Department of Medical Microbiology, Medical College, Qingdao University, Qingdao, China; ^3^Department of Physiology, Medical College, Qingdao University, Qingdao, China

**Keywords:** ghrelin, insulin resistance, obesity, T2DM, meta-analysis

## Abstract

**Background:** Ghrelin, a peptide mainly produced by stomach X-A cells. It plays a pivotal role in the regulation of food intake and energy metabolism, including glucose metabolism and insulin sensitivity. However, the correlation between circulating ghrelin levels and insulin resistance in obesity remained uncertain. This meta-analysis aimed to clarify the association between ghrelin and IR in obesity.

**Methods:** A systematic literature search was performed using PubMed, EMBASE, Cochrane Library and Web of Science until April 18, 2018 with the keywords “ghrelin” and “insulin resistance.” Two independent reviewers selected studies and assessed data. Subgroup analyses were performed to search for sources of heterogeneity. Funnel plots and Egger's test were used to detect publication bias. A random-effects model was used to calculate the pooled effect size.

**Results:** Ten studies with 546 participants were included in this meta-analysis. We found that ghrelin levels were negatively correlated with IR in obese individuals. (*r* = −0.31; 95% CI: −0.45 to −0.18). Subgroup analysis revealed that circulating ghrelin levels were significantly negatively correlated with IR in people with normal fasting blood glucose (FBG) (<6.9 mmol/dl) (*r* = −0.28; 95% CI: −0.47 to −0.09, *I*^2^ = 39.5%), while there was no relationship between circulating ghrelin levels and IR in the high FBG group (>6.9 mmol/dl) (*r* = −0.15; 95% CI: −0.33 to 0.03, *I*^2^ = 0.0%). Publication bias was insignificant (Egger's test: *P* = 0.425).

**Conclusion:** In obesity, circulating ghrelin levels were significantly negative correlated with insulin resistance in individuals with normal fasting blood glucose.

## Introduction

With the improvement of living standards, obesity has become a major threat to public health. The prevalence of obesity is increasing at an alarming rate worldwide. In 2015, about 2 billion people were overweight and one third of these were obese (Seidell and Halberstadt, 2015). There are many co-morbidities related to obesity that can lead to increasing morbidity and mortality, including type 2 diabetes mellitus (T2DM), cancers and cardiovascular disease (Guh et al., 2009). T2DM is a long-term metabolic disorder characterized by high blood sugar, insulin resistance (IR) and relative lack of insulin (DeFronzo et al., 2015). The common pathology of obesity and T2DM is IR. IR is a pathological condition in which cells are resistant to insulin, leading to high blood sugar. Beta cells in the pancreas increase secretion of insulin after sensing high sugar levels, further contributing to a high blood insulin levels. IR has been identified as a collective health problem (Sah et al., 2016). Solutions to IR can provide new therapeutic targets for both obesity and T2DM.

Ghrelin is a peptide of 28 amino acids that is an endogenous natural ligand for the growth hormone (GH) secretagogue receptor 1α. It is primarily produced by X-A cells in the stomach (Kojima et al., 1999; Date et al., 2000). In addition to the stomach, ghrelin also distributes in many other central or peripheral tissues, like hypothalamus, pituitary gland, lung, liver, kidney, endocrine pancreas, and adipose tissue (van der Lely et al., 2004). There are two forms of ghrelin, acylated ghrelin (AG) and des-acyl ghrelin (DAG). AG is acylated by the enzyme ghrelin-O-acyl transferase (GOAT) at amino acid serine 3, which is essential for its bioactivity (Zizzari et al., 2011). The main form of circulating ghrelin is DAG, occupied 80–90% to the total ghrelin (Pacifico et al., 2009). In addition to its basic function promoting GH release (Kojima et al., 1999), ghrelin regulates feeding (Nakazato et al., 2001), body weight (Tschöp et al., 2001; Ariyasu et al., 2002; Shiiya et al., 2002), and energy metabolism (Ledderose et al., 2011). Studies demonstrated that ghrelin was involved in glucose metabolism and participated in the regulation of insulin release and insulin sensitivity (Pinkney, 2014; Yada et al., 2014). It is reported that AG and DAG have opposite effect on insulin metabolism. AG stimulated glucose release, while DAG could counteract the stimulatory effect of AG (Broglio et al., 2004; Gauna et al., 2005). Gauna et al. demonstrated that AG induced IR, whereas a combination of DAG and AG improved insulin sensitivity (Gauna et al., 2004). These findings suggested that ghrelin may act as a new therapeutic target for IR.

Obesity changed circulating ghrelin profile, and ghrelin was associated with IR in obesity (Pacifico et al., 2009), while the relationship between ghrelin and IR is controversial in the population with obesity. A great number of studies indicated that there was a negative correlation between ghrelin and IR in obesity (Ikezaki et al., 2002; Pedrosa et al., 2011; Wadden et al., 2012; Al Qarni et al., 2017), while some studies obtained different results: In the study of Stepien et al., the ghrelin was negative with IR in the obesity plus insulin sensitivity group while positive with IR in the obesity plus insulin resistance group (Stepien et al., 2011). In the study of Hamed et al., the ghrelin was negative with IR in the simple obesity group while positive with IR in the obesity plus T2DM group (Hamed et al., 2011). The aim of this meta-analysis was to clarify the association between ghrelin and IR in obesity.

## Methods

Our meta-analysis was based on the Preferred Reporting Items for Systematic Reviews and Meta-analyses guidelines (Moher et al., 2015).

### Date sources and search strategy

A systematic literature search of PubMed, EMBASE, Cochrane Library and Web of Science was performed to April 18, 2018. The search used broadly defined medical subject headings including “ghrelin” and “insulin resistance.” In pubMed, the following free terms were searched: “GHRL protein,” “Ppghrelin,” “obestatin,” “appetite-regulating hormone,” “appetite regulating hormone,” “ghrelin-obestatin preprohormone,” “ghrelin obestatin preprohormone,” “motilin-related peptide precursor,” “motilin related peptide precursor,” “motilin-related peptide,” “motilin related peptide,” “gastric MLTRP,” “peptide precursor, motilin-related,” “precursor, motilin-related peptide,” “PpMTLRP,” “ghrelin precursor,” “precursor, ghrelin,” “insulin sensitivity,” “resistance, insulin,” “sensitivity, insulin.” The titles and/or abstracts were reviewed to exclude irrelevant studies, and the full texts of the remaining studies were read by two authors (C.Z, L.W) independently. Relevant studies were qualified after agreement was reached. In order to obtain a complete set of relevant studies, we used a search strategy combining two separate parts in the several searches. In addition, the reference lists of relevant studies were inspected manually to identify any papers that had been missed.

### Study selection and criteria

Studies were eligible if they met the following criteria: (1) conducted in humans; (2) studied obesity with or without T2DM; (3) used correlation coefficient analyses to report the association between circulating ghrelin (AG or DAG or total ghrelin, measured in plasma or serum) and IR assessed by the homeostasis model assessment of IR (HOMA-IR)(Matthews et al., 1985); (4) published in English; (5) original articles with ≥10 subjects. Exclusion criteria were as follows: (1) studies including obese individuals with accompany disease including metabolic syndrome, psychiatric disorders, cancer, stroke, severe hepatic or renal disease and acute cardiovascular events et al; (2) reviews, meta-analysis, case reports, letters and comments, meeting abstracts, and posters were also excluded.

### Data extraction and quality assessment

Data were extracted into a predefined standardized form by two authors (C.Z, L.W) who screened and assessed full texts. Any disagreements were resolved through discussion. The collected data included: study information (author, published year, country, study design, study population, number of men, and women); and subject characteristics (age, BMI, correlation coefficients, ghrelin type, fasting blood glucose, and blood samples).

The methodological quality of the included studies was evaluated by two authors (R.W, Y.L) independently using the eleven-point Agency for Healthcare Research and Quality (AHRQ) (Bindman, 2017) criteria. According to AHRQ criteria, article quality was assessed by the following (Supplementary Table A): A: defined the source of information; B: listed inclusion and exclusion criteria for exposed and unexposed subjects; C: indicated time period used for identifying patients; D: indicated that the subjects were consecutive if not population-based; E: indicated whether evaluators of subjective components of study were masked to other aspects of the status of the participants; F: described assessments undertaken for quality assurance purposes; G: explained patient exclusions from analysis; H: described how confounders were assessed and/or controlled; I: explained how missing data were handled in the analysis; J: summarized patient response rates and completeness of data collection; K: whether to clarify follow-up.

We used the New-castle-Ottawa Scale (NOS) to assess three items in case-control studies (Supplementary Table B): A: whether the definitions of obesity and T2DM were adequate; B: whether the cases were representative; C: whether the control group was from the same community; D: whether the control group subjects had a history of the disease; E: whether the design or analysis was comparable between cases and controls; F: whether ascertainment of exposure included secure records or structured interviews that were blind to case/control status; G: whether the method of ascertainment for cases and controls were identical; and H: whether the cases and controls showed identical non-response rates. Stars were assigned to each parameter ranging from 0 (the lowest) to 9 (the highest). Studies with a score ≥7 were considered to be of high quality, and other studies were classified as having moderate quality.

### Data synthesis and analysis

We estimated the associations between ghrelin and IR (HOMA-IR) using correlation coefficients and generated forest plots using a random-effects model. Correlation coefficients require Fisher's z transformation to calculate the relevant statistics. Meta-analyses generated variance and 95% CIs before converting them back to obtain the summary effect size (r). Heterogeneity was explored and quantified by the I^2^ statistic and values ≥50% were interpreted as having substantial heterogeneity. Publication bias was assessed by the Egger's regression asymmetry test and inspection funnel plots. All statistical analyses in this meta-analysis were carried out using Stata Software (Version 12.0).

## Results

### Literature search

**Figure 1:**
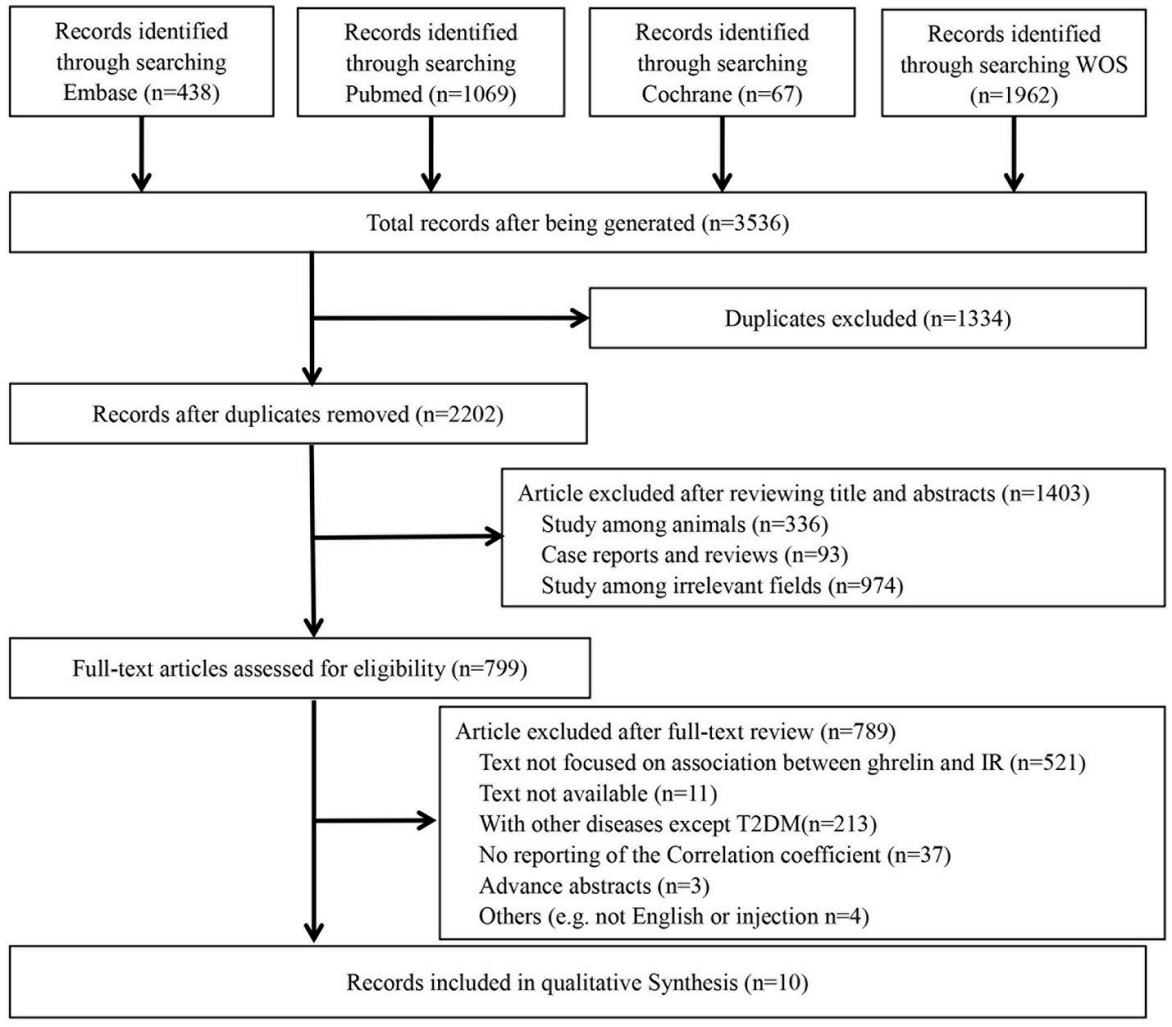
Flow chart of literature search.

The details of the search are shown in Figure 1. A total of 3,536 potential reports were filtered from four online databases, including PubMed, Embase, Cochrane Library and Web of Science. After duplication check, 1,334 studies were removed. After reviewing titles and abstracts, 1,403 ineligibles titles were removed. After reviewing full texts, 789 were removed. Finally, 10 studies (Ikezaki et al., 2002; Hamed et al., 2011; Homaee et al., 2011; Pedrosa et al., 2011; Stepien et al., 2011; Afify et al., 2012; Bellone et al., 2012; Wadden et al., 2012; Al Qarni et al., 2017; Methak et al., 2017) were included in this meta-analysis.

### Study characteristics

In the study of Stepien et al., the subjects were divided into two groups as obesity plus insulin sensitivity (IS) and obesity plus IR. In the study of Hamed et al., the subjects were divided into two groups as simple obesity and obesity plus T2DM. Thus, 10 studies with 12 groups were included in the meta-analysis. The selected 10 studies (12 groups) including 546 participants were published from 2002 to 2017. The characteristics of the studies are displayed in Table 1. The BMI of all the participants exceeded 27 kg/m^2^ except one study reporting that BMI in children was 22 kg/m^2^ [classified as obese (≥95th BMI percentile) according to the US Centers for Disease Control and Prevention] (Kuczmarski et al., 2000). Subjects in nine groups were simply obese; one study divided patients into two groups (obesity plus IS and obesity plus IR); and one study used one T2DM, two obese with T2DM corresponding to BMI. Three reports studied preteens, and nine studied adolescents and post-adolescents. Blood samples were obtained from serum in six groups, plasma in five groups and both in one group. Ghrelin levels were measured by enzyme linked immunos orbent assay (ELISA) in seven studies and three by radioimmunoassay (RIA), with one study unclear. In all studies, acylated and total ghrelin level were tested in five groups, and in two groups this was unclear. The sample size ranged from 19 to 108.

**Table 1:** Characteristics of included studies.

**Author. year**	**Country**	**Study design**	**Study population**	**Number (male/female)**	**Age**	**BMI**	**Correlation coefficient between Ghrelin (type) and IR**	**Blood glucose**	**Blood sample**
Afify et al., 2012	Egypt	Cross-sectional	Obese	29	49 ± 1.2	33.43 ± 1.65	−0.542 (TG)	84.2 mg/dL	Plasma
Al Qarni et al., 2017	Saudi Arabia	Cross-sectional	T2DM	97	–	32.5	−0.195 (AG)	7.8 mmol/L	Plasma
Bellone et al., 2012	Italy	Cross-sectional	Obese	58 (28/30)	9.8 ± 3.4	27.03 ± 0.62	−0.544 (AG)	–	Plasma
Hamed et al., 2011	Egypt	Cross-sectional	Obese	30 (13/17)	43.23 ± 7.48	36.12 ± 1.73	−0.077 (TG)	5.74 mg/dL	Serum
Hamed et al., 2011	Egypt	Case-control	Obese with T2DM	30 (12/18)	43.80 ± 4.64	35.30 ± 1.78	0.011 (TG)	16.74 mg/dL	Serum
Homaee et al., 2011	Iran	Cross-sectional	Obese	19 (19/–)	27.5 ± 5.8	31.03 ± 3.59	−0.48 (AG)	4.9 mmol/L	Plasma
Ikezaki et al., 2002	Japan	Cross-sectional	Obese	49 (38/11)	10.2 ± 2.8	28.0 ± 4.5	−0.3 (TG)	–	Plasma
Methak et al., 2017	Iraq	Case-control	Obese with T2DM	108 (49/54)	52.46 ± 0.82	34.03 ± 0.45	−0.46 (AG)	–	Serum
Pedrosa et al., 2011	Portugal	Cross-sectional	Obese	61 (21/34)	7~9	–	−0.284 (TG)	4.48 mmol/L	Plasma
Stepien et al., 2011	Poland	Cross-sectional	Obese with IS	19 (4/15)	53.0 ± 13.19	33.19 ± 3.13	−0.4947 (AG)	5.46 mmol/L	Serum
Stepien et al., 2011	Poland	Cross-sectional	Obese with IR	18 (7/11)	49.56 ± 14.16	34.77 ± 3.52	0.1723 (AG)	5.72 mmol/L	Serum
Wadden et al., 2012	Canada	Cross-sectional	Obese	28	23.25 ± 0.49	29.10 ± 0.92	−0.092 (AG)	5.28 mmol/L	Serum

*Data presented as means ± SEM. T2DM, type 2 diabetes mellitus. IR, insulin resistance. AG, acylated ghrelin. TG, total ghrelin*.

### Overall analysis

**Figure 2:**
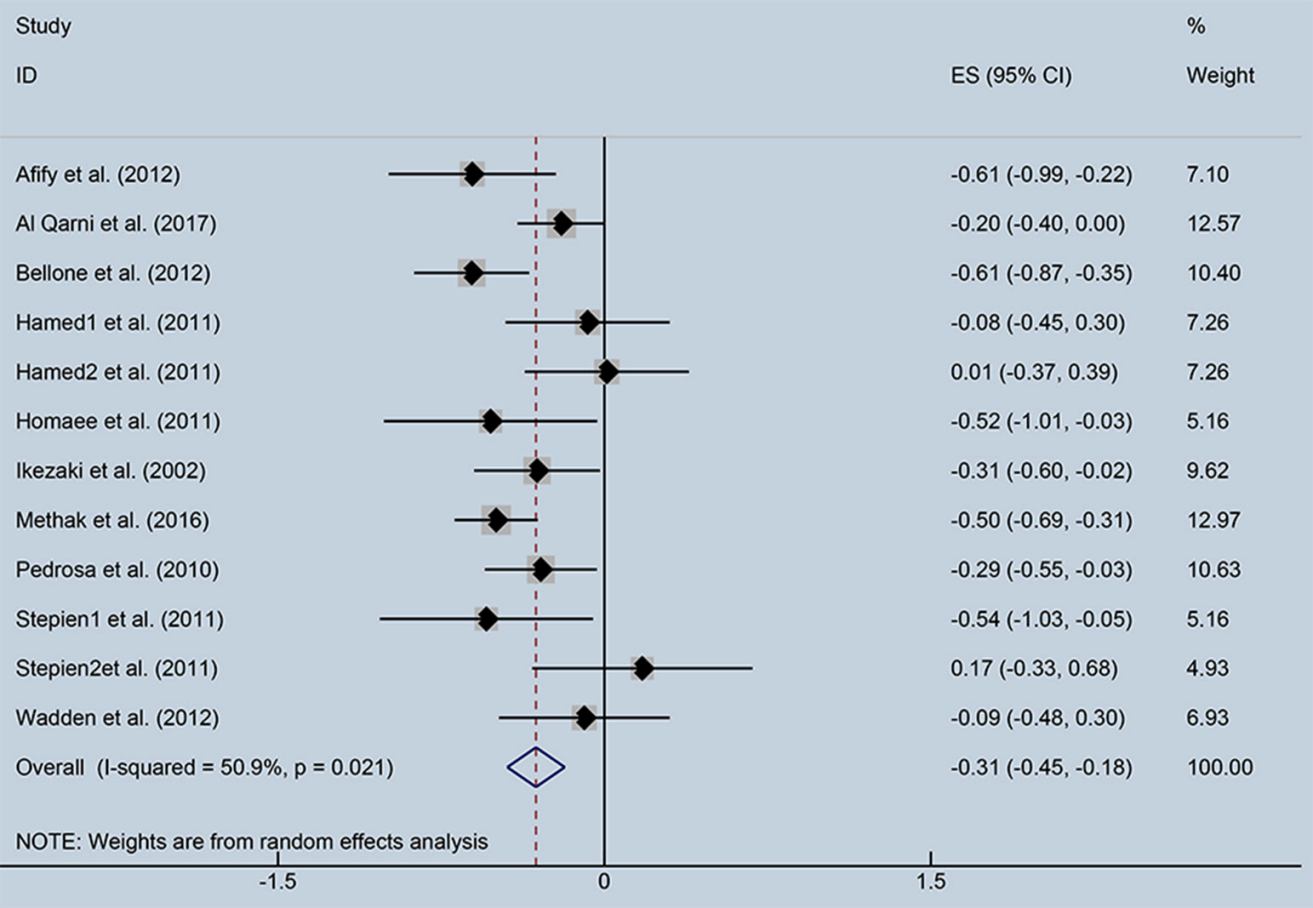
Forest plot showing the effect size of correlation coefficients between circulating ghrelin and insulin resistance. CI, Confidence interval. Summary estimates were analyzed using a random-effects model.

**Figure 3:**
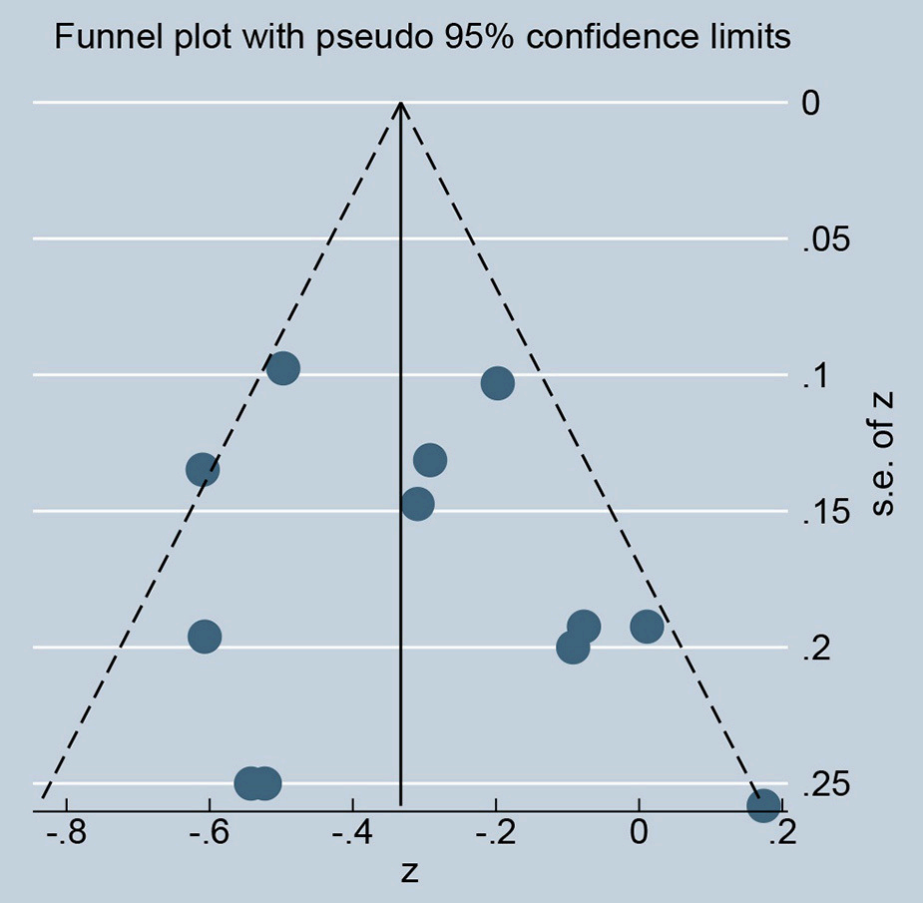
The funnel plot of the publication bias.

As showed in Figure 2, ghrelin levels were significantly inversely correlated with IR (*r* = −0.31, 95% CI: −0.45 to −0.18). The *r*-value showed insignificant heterogeneity by a random effect model (*I*^2^ = 50.9%, *P* = 0.021). Publication bias was evaluated and was considered insignificant (Supplementary Figure; Egger' test: *P* = 0.425). The funnel plot is shown in Figure 3.

### Subgroup analysis

**Figure 4:**
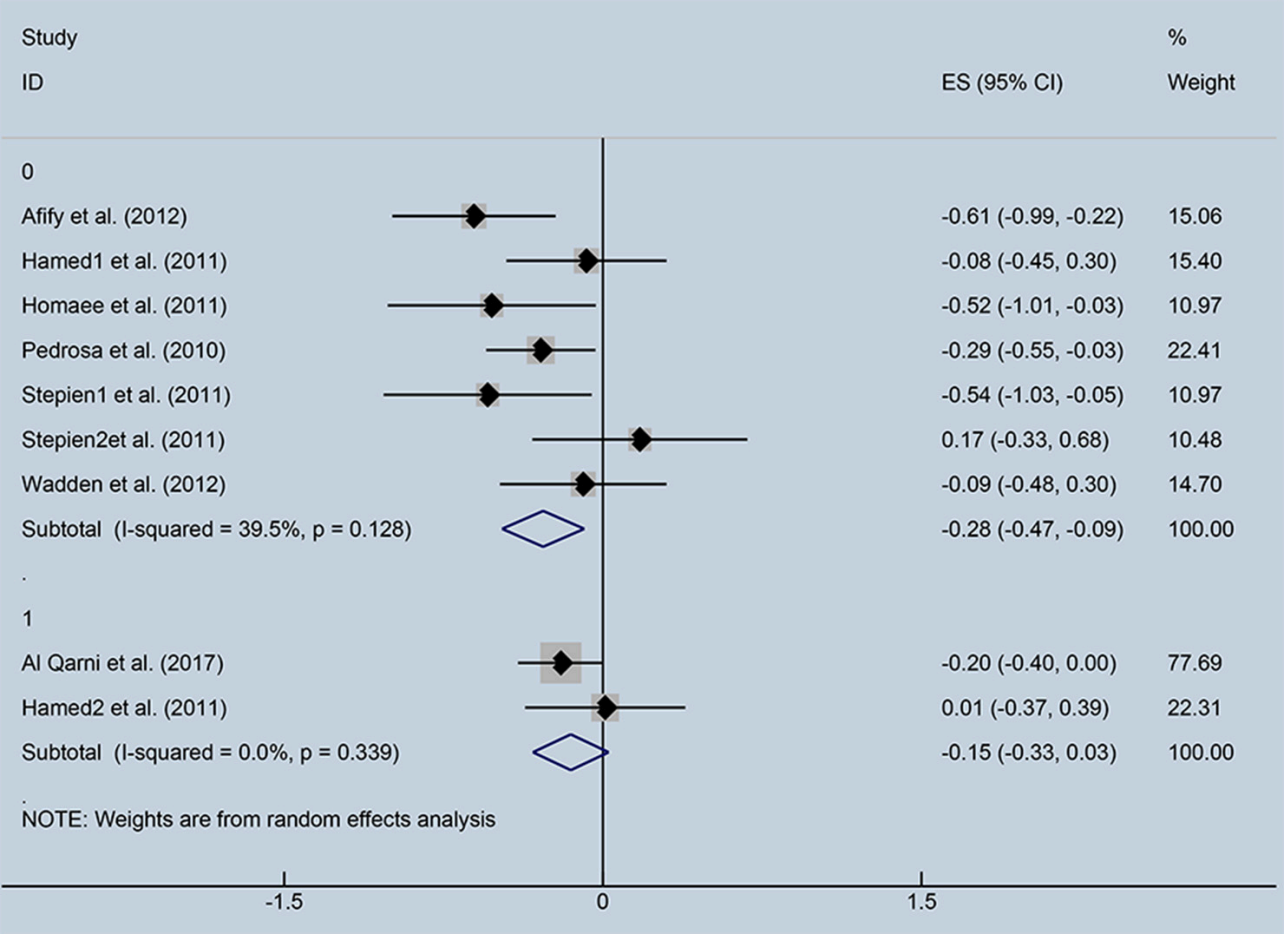
Forest plot of correlation coefficients between circulating ghrelin and insulin resistance based on fasting blood glucose levels. CI, Confidence interval. Summary estimates were analyzed using a random-effects model. Afify et al., Hamed et al., Homaee et al., Pedrosa et al., Stepien et al., Stepien et al., Wadden et al. represented data from people with normal fasting blood glucose; Al Qarni et al., Hamed et al. represented data from people with high fasting blood glucose.

Subgroup analysis was performed to investigate the factors that influenced heterogeneity. We found that the correlation index between ghrelin and IR depended on FBG levels. Therefore, all included studies were divided into two subgroups: normal FBG (<6.9 mmol/dl) and high FBG (>6.9 mmol/dl), (Figure 4). Seven studies with normal FBG showed negative correlations between ghrelin and IR (*r* = −0.28, 95% CI: −0.47 to −0.09, *I*^2^ = 39.5%, *P* = 0.128). Two studies were divided into high FBG group, and the analysis showed that there was no relationship between circulating ghrelin levels and IR (*r* = −0.15, 95% CI: −0.33 to 0.03, *I*^2^ = 0.0%, *P* = 0.339). FBG in three studies was unspecified.

We carried out other subgroup analyses classified by ghrelin types (acylated, total or unspecified), age (adolescence, none adolescence or unspecified), BMI (obesity, excessive obesity, or unspecified) and blood samples (plasma or serum) (Table 2). These factors gave no statistical significance or reduced heterogeneity in the selected studies. Therefore, more information needs to be obtained for further analysis.

**Table 2:** Summary risk estimates of correlations between IR and ghrelin.

	**Groups**	**Participants**	**Random effects r (95% CI)**	**I2 (%)**	**P for heterogeneity**
Overall	12	546	−0.31(−0.45,−0.18)	50.9	0.021
Subgroup analysis					
**GHRELIN TYPE**					
Acylated	5	310	−0.39(−0.58,−0.19)	59.4	0.043
Total	5	199	−0.26(−0.44, −0.08)	35.3	0.186
Unspecified	2	37	−0.19(−0.89, 0.51)	74.8	0.046
**AGE**					
Adolescence	3	168	−0.41(−0.61, −0.20)	42.3	0.177
None adolescence	8	281	−0.28(−0.49, −0.08)	57.1	0.022
Unspecified	1	97	−0.20(−0.40, 0.00)		
**BMI**					
Excessive obesity	7	331	−0.27(−0.47, −0.07)	62.0	0.015
Obesity	4	154	−0.39(−0.62, −0.16)	44.3	0.145
Unspecified	1	61	−0.29(−0.55, −0.03)		
**BLOOD SAMPLE**					
Plasma	6	313	−0.39(−0.54, −0.23)	39.8	0.140
Serum	6	233	−0.19(−0.44, 0.05)	62.7	0.020

*CI, Confidence interval*.

## Discussion

To our knowledge, this was the first meta-analysis to investigate the relationship between ghrelin and IR in population with obesity. Ten original studies and 17 groups of data were included. Our analysis demonstrated that the level of ghrelin was negatively associated with IR in obese populations with normal FBG. This finding supports the hypothesis that ghrelin may be a new therapeutic target for obesity. By monitoring the level of ghrelin, people can predict the possibility of obesity. Besides, ghrelin may act as a therapeutic factor to modulate insulin resistance in obesity and T2DM.

In 2002, Al Qarni et al. found that fasting plasma ghrelin levels were negatively correlated with IR in obese children and adolescents (Al Qarni et al., 2017). Several studies reached the same conclusion (Ikezaki et al., 2002; Pedrosa et al., 2011; Pinkney, 2014), including not only children and adolescents, but also adults. However, Stepien et al. found that fasting plasma ghrelin levels were negatively correlated with IR in obese people with IS, while there was a positive correlation between fasting ghrelin and IR in obese people with IR (Stepien et al., 2011). According to our meta-analysis, we found that circulating ghrelin levels were negatively related to IR in obesity, but heterogeneity did exist among included studies. In order to identify the source of heterogeneity, we performed numerous subgroup analyses. In subgroup analysis based on various FBG levels, heterogeneity was reduced, indicating that FBG was an important factor. In the high FBG group, there was no correlation between ghrelin level and IR, perhaps because only two studies were in the group and the sample number in the high FBG groups was inadequate. Furthermore, once the FBG was higher than normal levels, the individual may develop severe diabetes or obesity-associated diabetes. In that case, ghrelin levels may be affected (Cruz-Dominguez et al., 2014), and the relationship between circulating ghrelin and IR may be different. Thus, further studies are needed.

We analyzed the source of heterogeneity using several other factors including ghrelin types, age, BMI, and blood samples. Nevertheless, the results suggested that the correlation may be not affected by these factors, and further clinical trials are needed to confirm these conclusions. It should be noticed that most of the included studies were from Europe or Africa, where obesity are less prevalent compared to the US or India. We tried but failed to obtain more studies from obesity prevalent countries and because of the limited number of studies from these countries, we were unable to conduct subgroup analysis based on obesity prevalence. The heterogeneity in our study might be related to the lower prevalence, however, the significance generated from our study suggests that it is highly possible that the association between circulating ghrelin and IR is the same in these obesity prevalent countries. And considering the high obesity prevalence in these countries, application of monitoring ghrelin in routine obesity screening is possible, and should be taken into consideration for prediction and prevention of obesity in an early stage. For that reason, it is recommended to conduct more related clinical trials in countries with higher obesity prevalence to verify our findings.

Ghrelin take part in glucose metabolism (Ledderose et al., 2011; Yada et al., 2014; Poher et al., 2018), and may function as a biomarker for IR in obesity, while the mechanisms remain undetermined. Ghrelin is primary produced by stomach, while the pancreas is also an important production site for ghrelin. There are ghrelin messenger RNA (mRNAs) expressed in the pancreatic α-cells (Date et al., 2002), and ghrelin GOAT is highly expressed in pancreatic islets (An et al., 2010), suggesting that the pancreas not only produces ghrelin but also is important for ghrelin function. Date et al. reported that ghrelin stimulated insulin secretion by increasing cytosolic free Ca2^+^ concentrations in β-cells (Date et al., 2002). These findings indicated that ghrelin may alleviate insulin resistance in an endocrine and/or paracrine manner. Barazzoni et al. suggested that ghrelin ameliorated insulin resistance by activating muscle AKT-GSK signaling and subsequently increasing tissue glucose utilization (Barazzoni et al., 2007). In obesity, treatments targeting the reduction of blood glucose, or increasing insulin sensitivity can be carried out while monitoring ghrelin levels.

There are some limitations to the current study. Sample number of some included studies was small causing significant heterogeneity in our meta-analysis, therefore, additional large-scale studies are needed to increase the quality of studies. A mass of literature search was provided in PubMed, EMBASE, Cochrane Library, and Web of Science databases, therefore the language restriction may have augmented the risk of publication bias. Few studies explored whether improving IR therapies can change the association between ghrelin and IR, therefore we could not demonstrate the effect of anti-IR therapies based on current evidence. Furthermore, there are several methods of assessing the degree of IR, of which the hyperinsulinemic euglycemic clamp technique is regarded as the gold standard. However, in clinical application, this technique is not universally applicable. We chose only the index of IR (HOMA-IR) to evaluate IR and to exclude some relevant studies that did not report the correlation coefficient between ghrelin and HOMA-IR, causing some bias on our results.

In summary, we found that ghrelin was negatively associated with IR in obesity. In order to strengthen this meaningful association, additional large-scale studies that measure both ghrelin and IR are needed to determine whether ghrelin administration can ameliorate IR in obesity. Despite some limitations in our study, we believe that this meta-analysis has significance for follow-up research to explore the possible pathophysiological mechanisms underlying this relationship.

## Author contributions

C-SZ and L-XW designed the program, searched and reviewed studies, were in charge of the manuscript. RW and YL assessed studies, extracted data, wrote part of the manuscript. L-MS extracted data, wrote part of the manuscript. J-HY reviewed and edited the manuscript. BW and JD directed the project, contributed to discussion, reviewed and edited the manuscript. As the corresponding authors, BW and JD had full access to all the data in the study and had final responsibility for the decision to submit for publication. C-SZ and L-XW contributed equally to this work.

### Conflict of interest statement

The authors declare that the research was conducted in the absence of any commercial or financial relationships that could be construed as a potential conflict of interest.
