# Supplementary material for: The Correlation Between Circulating Ghrelin and Insulin Resistance in Obesity: A Meta-Analysis
Source: Front Physiol. 2018 Sep 21;9:1308. doi: 10.3389/fphys.2018.01308 (PMC6160589; doi:10.3389/fphys.2018.01308)
Supplement: Supplementary file 1 [file Table_1.pdf]

A Quality assessment of included Cross-Sectional studies

study	A	B	C	D	E	F	G	H	I	J	K	Total
Afify *et al*.	1	1	0	1	1	0	1	1	0	0	0	6
Al Qarni *et al*.	1	1	1	1	1	0	1	1	0	0	0	7
Bellone *et al*.	1	1	0	1	1	0	1	1	0	0	0	6
Homaee *et al*.	1	1	0	1	1	0	1	1	0	1	0	7
Ikezaki *et al*.	1	1	0	0	1	0	1	0	0	0	0	4
Pedrosa *et al*.	1	1	0	1	1	1	1	1	1	1	1	10
Stepien et al	1	1	0	1	1	1	1	0	0	1	0	7
Wadden *et al*.	1	1	0	1	1	1	1	1	0	0	0	7

A: Define the source of information (survey, record review); B: List inclusion and exclusion criteria for exposed and unexposed subjects (cases and controls) or refer to previous publications; C: Indicate time period used for identifying patients; D: Indicate whether or not subjects were consecutive if not population-based; E: Indicate if evaluators of subjective components of study were masked to other aspects of the status of the participants; F: Describe any assessments undertaken for quality assurance purposes (e.g., test/retest of primary outcome measurements); G: Explain any patient exclusions from analysis; H: Describe how confounding was assessed and/or controlled; I: If applicable, explain how missing data were handled in the analysis; J: Summarize patient response rates and completeness of data collection; K: Clarify what follow-up, if any, was expected and the percentage of patients for which incomplete data or follow-up was obtained.

B Quality assessment of included case-control studies

study	A	B	C	D	E	F	G	H	Total
Hamed *et al*.	1	1	0	1	1	1	1	0	6
Methak *et al*.	1	1	0	1	1	1	1	0	6

A: The case definition is adequate; B: Representativeness of the cases; C: Community controls; D: No history of disease in controls; E: Comparability of cases and controls on the basis of the design or analysis; F: Ascertainment of exposure include secure record or structured interview where blind to case/control stetus; G: Same method of ascertainment for cases and controls; H: Same non-response rate for both groups.
