# Supplementary figures and images for: The Correlation Between Circulating Ghrelin and Insulin Resistance in Obesity: A Meta-Analysis
Source: Front Physiol. 2018 Sep 21;9:1308. doi: 10.3389/fphys.2018.01308 (PMC6160589; doi:10.3389/fphys.2018.01308)

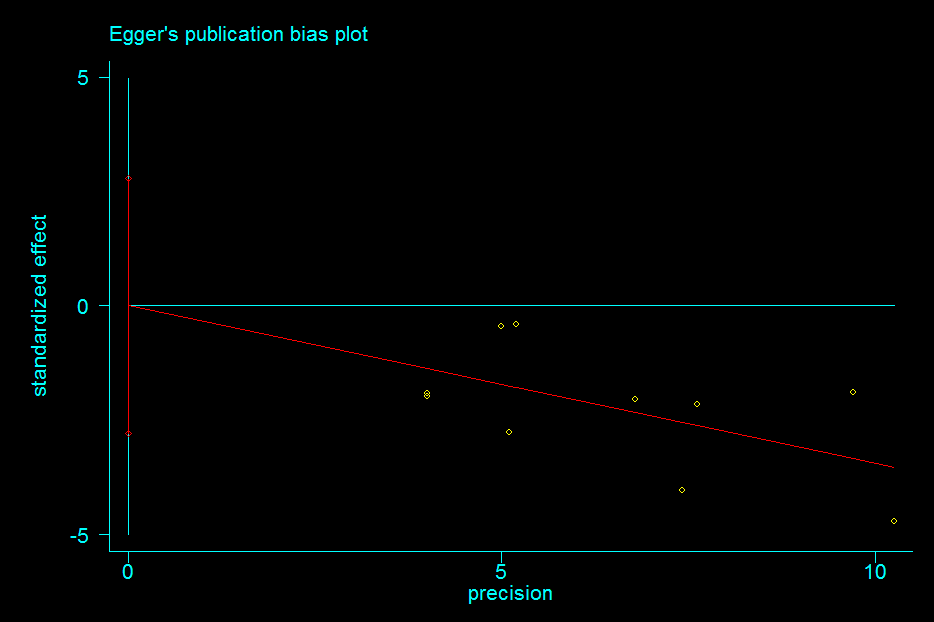

Supplement: Supplementary file 2 [file Image_1.TIF]
